# Health trajectories reveal the dynamic contributions of host genetic resistance and tolerance to infection outcome

**DOI:** 10.1098/rspb.2015.2151

**Published:** 2015-11-22

**Authors:** Graham Lough, Ilias Kyriazakis, Silke Bergmann, Andreas Lengeling, Andrea B. Doeschl-Wilson

**Affiliations:** 1 Genetics and Genomics Division, The Roslin Institute and R(D)SVS, University of Edinburgh, Edinburgh, UK; 2 Infection and Immunity Division, The Roslin Institute and R(D)SVS, University of Edinburgh, Edinburgh, UK; 3 School of Agriculture, Food and Rural Development, Newcastle University, Newcastle upon Tyne, UK; 4 Department of Infection Genetics, Helmholtz Centre for Infection Research, Braunschweig, Germany

**Keywords:** resistance, tolerance, trajectories, complex traits genetics, infection dynamics, host response strategies

## Abstract

Resistance and tolerance are two alternative strategies hosts can adopt to survive infections. Both strategies may be genetically controlled. To date, the relative contribution of resistance and tolerance to infection outcome is poorly understood. Here, we use a bioluminescent *Listeria monocytogenes* (*Lm*) infection challenge model to study the genetic determination and dynamic contributions of host resistance and tolerance to listeriosis in four genetically diverse mouse strains. Using conventional statistical analyses, we detect significant genetic variation in both resistance and tolerance, but cannot capture the time-dependent relative importance of either host strategy. We overcome these limitations through the development of novel statistical tools to analyse individual infection trajectories portraying simultaneous changes in infection severity and health. Based on these tools, early expression of resistance followed by expression of tolerance emerge as important hallmarks for surviving *Lm* infections. Our trajectory analysis further reveals that survivors and non-survivors follow distinct infection paths (which are also genetically determined) and provides new survival thresholds as objective endpoints in infection experiments. Future studies may use trajectories as novel traits for mapping and identifying genes that control infection dynamics and outcome. A Matlab script for user-friendly trajectory analysis is provided.

## 1. Background

Two alternative host response strategies to pathogen challenge contribute to survival: *resistance*, defined as the ability of a host to limit or inhibit pathogen replication, thus reducing infection severity [1]; and *tolerance*, defined as the ability of an infected host to limit the impact of infection on fitness or health. Tolerance mechanisms reduce or prevent damage associated with pathogen challenge, but have no direct impact on the pathogen itself [1–4]. In addition, tolerance is an important mechanism for the coevolution of symbiotic interactions between beneficial commensal microbes and the host, which has long been recognized in both plants and animals [5,6]. As host strategies, both resistance and tolerance may be genetically determined [2,7,8]. Assessment of their relative contribution to survival requires quantitative estimates of resistance and tolerance based on empirical evidence. Resistance may be defined as the inverse of infection severity, conventionally quantified by measures of within-host pathogen burden. Obtaining quantitative estimates of tolerance has proved difficult in practice, owing to its statistical definition as reaction norm of health with respect to changes in pathogen burden [2,9,10] and the high frequency of measurements associated with constructing and analysing reaction norms [10–13]. Although conceptually defined at the individual level, quantitative tolerance estimates can usually only be obtained at the level of groups of (related) individuals, which constitutes a major limitation to unravelling the host genetic regulation of tolerance [10–12].

**Figure 1.**
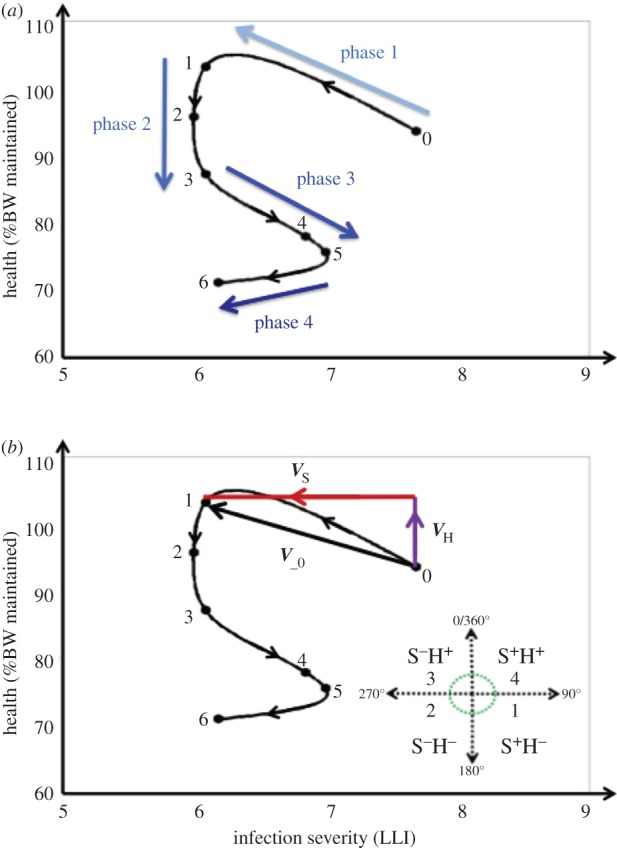
An infection severity–health (SH) trajectory for an individual mouse. (*a*) Illustration of trajectory phases from start of infection to death. A trajectory for an individual mouse was produced by plotting longitudinal pairwise measurements of body weight (BW) and infection severity (bacterial load measured by log-transformed light intensity plus one; LLI) in a two-dimensional space, and following their progression over time. The graph also shows the four characteristic phases of infection associated with distinct changes in infection severity and health (indicated by arrows), as described in the text. (*b*) Illustration of trajectory vectors and resulting sequences. The trajectory vector ***V***__0_ with components *V*_H_ (change in %BW) and *V*_S_ (change in infection severity) represents simultaneous change in infection severity (decrease; S^−^) and health (increase; H^+^) between 0 and 1 dpi. The bottom right panel shows the four quadrants (S^+^H^−^, S^−^H^−^, S^−^H^+^, S^+^H^+^) in the SH plane specifying the direction of a trajectory vector, together with the associated sequence numbers (1–4). Each trajectory is mapped to a sequence comprising 14 numbers, representing the directions of the trajectory vectors at 14 consecutive daily intervals, and with the sequence number 0 indicating death. The trajectory of the individual depicted in this figure corresponds to the sequence {3,2,2,1,1,2,0,0,0,0,0,0,0,0} (i.e. it died after dpi 6).

These static definitions of resistance and tolerance, and the limitation of estimating tolerance at the group level, cannot further our understanding of the relative contribution of resistance and tolerance to individual survival, which is likely to change over the time course of infection [14]. Recently, individual health trajectories have been introduced as potentially powerful tools to capture the dynamic nature of infection and its impact on health in individual hosts [14,15]. Trajectories are constructed by plotting individual measurements of infection severity (e.g. pathogen burden) against health in two-dimensional space at different stages of the infection. Following this pairwise progression over time produces a trajectory that illustrates the dynamic interplay of resistance and tolerance mechanisms by describing how changes in within-host pathogen burden are associated with changes in health throughout the infection period, not currently captured by static definitions of resistance and tolerance (figure 1*a*). We suggest that using trajectories as an alternative to conventional statistical analysis of resistance and tolerance will help describe individuals' infection paths towards a specific outcome (e.g. death or survival), and reveal critical stages of the infection associated with the greatest impact on health or fitness. It has been postulated that infection trajectories can be classified into distinct trajectory types [14,15] that may be linked to genetic background of the host [14]. It may thus be possible to map host genotypes to specific trajectory types, and target these for genetic improvement of host response to infections [15].

Although proved to be powerful on conceptual grounds, the use of trajectories to study host response to infection has not previously been supported by experimental data [14,15]. Their wider application in infectious disease research has been hampered by the lack of statistical methods for quantitative trajectory analyses [12]. Trajectories often display loops (figure 1*a*), which implies that they cannot be represented by mathematical functions, and are thus not amenable to conventional statistical models.

In this study, we develop a novel statistical framework for quantitative trajectory analysis, making use of non-invasive bioluminescent imaging tools to analyse the time course of listerial infection in four inbred mouse strains. *Listeria monocytogenes* (*Lm*) is a Gram-positive, facultative intracellular bacterium that causes food-borne infections in animals and humans. *Lm* is responsible for the life-threatening disease listeriosis in elderly and immunocompromised individuals [16,17]. In healthy individuals, *Lm* infections are usually self-limited but can cause acute, febrile gastroenteritis [18]. Inbred mouse strains differ substantially in their apparent susceptibility to listeriosis, through contributions of multiple genetic loci [19–21], but genetic variation in tolerance to *Lm*, and the relative contributions of resistance and tolerance to survival, are currently unknown. We use this model system (i) to determine whether there is genetic variation in tolerance to *Lm,* and whether mouse strains rank similarly in terms of resistance and tolerance, (ii) to study the kinetic infection severity–health (SH) relationships using trajectory analysis, and their association with survival, and (iii) to assess whether different host genotypes map on to distinct trajectory types.

## 2. Material and methods

### (a) Mice

The data were obtained from *Lm* infection challenge experiments of 84 mice from four genetically diverse inbred mouse strains as outlined by Bergmann *et al.* [22]. Briefly, female mice aged between 9 and 10 weeks from the strains A/J, BALB/cJ (BALB) and C57BL/6 J (B6 J), and C3HeB/FeJ (C3H) were orally infected with bioluminescent *Lm* as described below. The inbred mouse strains were selected because of known differences in resistance to listeriosis development, similar mature body weights, and for their suitability for *in vivo* bioluminescence imaging (BLI). All mice were subjected to BLI or analysed for bacterial organ loads. On 1, 3, 5 and 7 days post-infection (dpi), 3–8 mice per strain were sacrificed to measure colony forming units (CFU) of *Lm* from organ homogenates [22]. This enabled assessment of the spread of *Lm* to different internal organs, and to calibrate the infection severity measures obtained by the BLI analysis (see below). Ten mice per strain were maintained after inoculation until 14 dpi, or until they had to be euthanized due to reaching humane endpoints of infection severity. This was the case for all mice from strain C3H, 80% of A/J mice and 40% of BALB mice, which were all euthanized between 5 and 7 dpi due to onset of clinical signs of advanced listeriosis, according to established protocols and approved animal welfare regulations [22]. All mice were housed under specific-pathogen-free conditions. At the start of the experiment, all mice had reached mature body weights. Thus, any changes in body weight (BW) post-infection were assumed to be a direct consequence of the infection challenge.

### (b) Infection protocol

Prior to challenge with *Lm*, the mice were acclimatized for one to two weeks in the facility. On the day prior to infection, the mice were starved overnight, with drinking water replaced with carbonate buffered water. The next day, mice were intragastrically challenged with 5 × 10^9^ CFU *Lm* EGDe-InlA-mur-lux, an internalin A (*inlA*) modified strain of *Lm* as previously described [22,23]. After infection challenge mice had ad libitum access to both food and water.

### (c) Measurement of infection severity and health

In line with the literature, resistance was quantified as an inverse measure of infection severity [2], defined here in terms of log-transformed measures of light intensity (LLI) obtained daily from bioluminescent *in vivo* imaging (see electronic supplementary material, text S1). Higher LLI values correspond to higher *Lm* loads, which is indicative of higher infection severity [19].

As *Lm* infection in adult mice causes a significant drop in BW, BW was used as an indicator of impact of the infection on health. BW was recorded for each individual mouse immediately prior to infection, and daily post-infection over the 14-day duration of the experiment. The impact of infection on health at a particular dpi was then represented as percentage of BW (%BW) loss at that day from the initial BW at 0 dpi, and %BW maintained at that day was considered as the daily indicator for health.

### (d) Conventional statistical analysis of resistance and tolerance

The statistical analysis used data only from the 40 mice that had not been analysed prior to 14 dpi for CFU counts, as only these provided information about the association of resistance and tolerance to survival. Data were analysed with the SAS statistical package (2010, v. 9.3) using procedure proc MIXED.

#### (i) Estimating resistance and tolerance based on peak infection severity and minimum health

In accordance with Råberg *et al.* [2], we defined resistance in terms of maximum infection severity, here represented by peak LLI levels over the two-week observation period. Tolerance estimates were obtained accordingly based on maximum infection severity (peak LLI) and minimum health (maximum %BW loss) achieved during the observation period.

To assess genetic variation in resistance, a linear mixed model was used with peak LLI as the dependent variable, and mouse strain, binary survival outcome (succumbed to infection within 14 dpi, termed ‘non-surviving’; did not succumb to infection, termed ‘surviving’), and their interactions as fixed effects.

In line with existing studies of tolerance genetics [2,13], an analysis of covariance (ANCOVA) was used to assess genetic variation in tolerance. The ANCOVA was performed using maximum %BW loss as dependent variable and peak LLI as independent variable, and mouse strain, survival outcome and the corresponding interactions as fixed effects. The intercept was fixed at zero, corresponding to zero %BW loss in the absence of infection. The ANCOVA slope coefficients resulting from regressing individual health measures against infection severity provide group estimates of tolerance, where steeper negative slopes correspond to less tolerant groups. Differences between these slope estimates based on the *F*-test statistics for strain-by-infection severity interaction thus provide evidence for genetic variation in tolerance.

#### (ii) Assessing the sensitivity of resistance and tolerance estimates to time of measurement

To determine the sensitivity of resistance and tolerance estimates to the timing of measurement, we replaced the extreme measures of peak LLI and maximum %BW loss with daily measures of LLI and %BW loss to obtain daily least square means (LSM) for infection severity (inverse of resistance) and tolerance slope for every mouse strain by survival outcome using the repeated measurement models as outlined in the electronic supplementary material, text S2.

### (e) Infection severity—health trajectories

Infection SH trajectories were generated by plotting the 14 daily health measurements (represented by %BW maintained) against the corresponding infection severity measures (LLI) recorded until 14 dpi for each individual, or until time of death if infection-dependent euthanasia occurred prior to 14 dpi. Successive scatter points were connected using the spline curve and, for illustrative purposes, smoothed using the SM30 smoothing procedure in SAS. Figure 1*a* shows an example of a trajectory; individual trajectories of all mice in consideration are presented in the electronic supplementary material, text S3.

#### (i) Trajectory comparison and numerical representation

Trajectories were first visually inspected to determine common features and differences related to levels and timing of simultaneous changes in infection severity and health. ‘Bad neighbourhoods’, associated with subsequent death due to infection, were identified in the two-dimensional phase plane by simply overlaying trajectories of surviving mice and those that succumbed to infection.

For statistical comparison of trajectories associated with different individuals, trajectories were mapped to numerical sequences, which were constructed as follows: first, for each individual trajectory, daily two-dimensional vectors 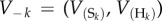 were produced as shown in figure 1*b*, where 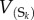 represents the change in infection severity (in LLI units) from day *k* − 1 to day *k*, and 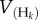 represents the corresponding change in health (%BW change). The magnitude of *V*_ *_k_* (i.e. *V_k_*) given by
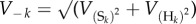
describes the rate of change in the two-dimensional host state between days *k*−1 and *k* (figure 1*b*). The direction of *V*_−*k*_ (determined by the signs of both vector components) indicates whether an increase or decrease in infection severity (S^+^/S^−^) between days *k*−1 and *k* is associated with a simultaneous improvement or deterioration in health (H^+^/H^−^). Four possible sign combinations give rise to four SH categories (1 = S^+^H^−^, 2 = S^−^H^−^, 3 = S^−^H^+^ and 4 = S^+^H^+^) according to which quadrant in the SH plane the trajectory vector faces (figure 1*b*). To reduce the impact of measurement noise in the statistical analysis, S^+^ or H^−^ were only assigned if infection intensity had increased by more than 0.1 LLI units, and BW had dropped by more than 2% compared with the last measurements, respectively. Otherwise, changes in either direction were assigned to S^−^ and H^+^, respectively. Finally, stringing the 14 daily consecutive SH combinations together generated an SH time series for each individual represented by a sequence of numbers between 0 and 4, where 0 indicates death of the host and 1–4 refer to the different combinations of simultaneous changes in infection severity and health as specified above (figure 1*b*).

#### (ii) Statistical analysis of trajectory sequences

Representing trajectories by numerical sequences allowed quantitative comparison of trajectories associated with different individuals. For this purpose, Hamming distances between all pairs of individual trajectory sequences, describing the proportions of non-zero sequence elements that differed between two sequences, were calculated. To prevent sequences associated with individuals who succumbed to infection within the 14-day observation period being assigned shorter distances, sequences were truncated to the last time point where both individuals of the pair in consideration were still alive.

In order to determine whether the 40 trajectories could be classified into few distinct types depending on their patterns as had been proposed previously [14,15], cluster analysis was carried out using the ‘clusterdata’ function in Matlab (v. R2013b), with the truncated Hamming distances as measure of similarity, and the weighted average distance as distance metric between clusters. The number of maximum clusters specified was 2, 4 and 6. Resulting clusters were visualized using BioLayout Express 3D [24].

Furthermore, a permutation test (in which trajectory sequences were randomized) was applied to test statistically significant differences between (truncated) trajectory sequences belonging to different clusters, mouse strains or survival groups. The permutation test assessed whether truncated Hamming distances between any two groups were on average significantly larger than the corresponding within-group distances. The corresponding Matlab script for generating sequences, calculating Hamming distances, and performing cluster analysis and permutation tests is provided in the electronic supplementary material, text S5.

## 3. Results

Infection was established in all mice, as indicated by high levels of LLI, and all mice experienced a drop in BW at a certain stage of infection, although at varying levels and duration. None of the mice were able to clear the infection within the 14-day experimental infection period. All B6J mice survived the infection period, whereas all C3H mice succumbed to infection within 6 dpi. There was within-strain variation in survival outcome for A/J and BALB mice: two A/J mice and six BALB mice survived until the end of the experimental observation period. This led to the following six mouse strains by survival outcome groups: C3H, B6J, A/J non-survivors, A/J survivors, BALB non-survivors and BALB survivors.

### (a) Estimates of resistance and tolerance

#### (i) Estimates of resistance and tolerance based on peak infection severity and minimum health

**Figure 2.**
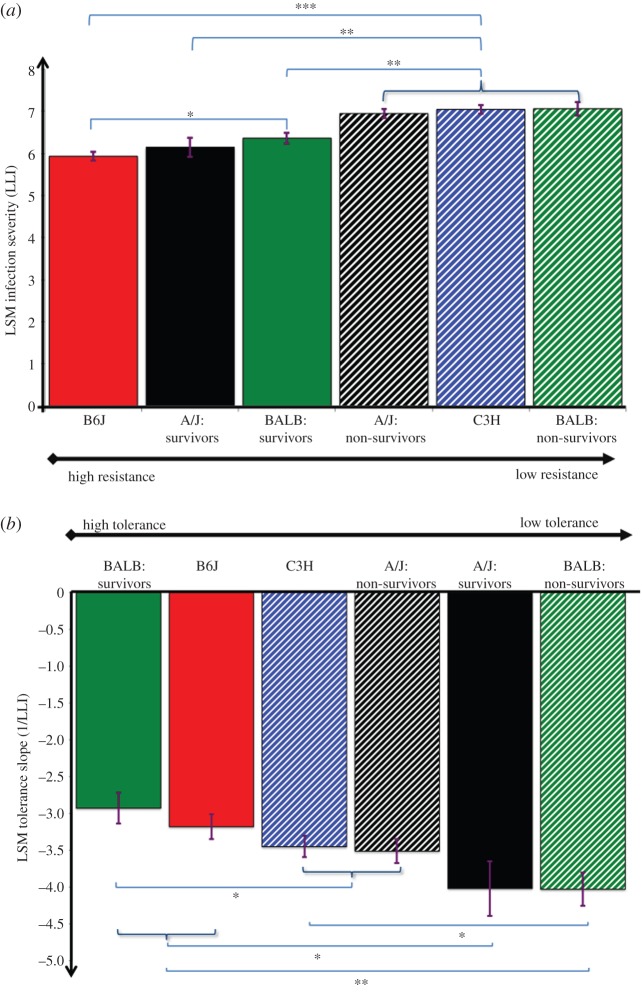
Least square mean (LSM) estimates for resistance and tolerance based on measures of maximum infection severity and minimum health. (*a*) LSM resistance quantified as the inverse of peak infection severity (LLI). (*b*) LSM tolerance (defined as regression slope based on regressing maximum %BW loss against LLI). Different mouse inbred strains are indicated by different colours and mouse strains or defined subgroups that succumbed to infection are indicated by dotted lines. LSM estimates (standard errors, s.e.) for LLI ordered from least to most resistant were: BALB non-survivors: 7.06(0.16); C3H: 7.04(0.10); A/J non-survivors: 6.95(0.11); BALB survivors: 6.36(0.13); A/J survivors: 6.14(0.23); B6J: 5.93(0.10). LSM tolerance estimates (s.e.) ordered from least to most tolerant were: BALB non-survivors: −4.03(0.23); A/J survivors: −4.02(0.37); A/J non-survivors: −3.51(0.17); C3H: −3.45(0.15); B6J: −3.18(0.17); BALB survivors: −2.93(0.21). Stars indicate statistically significant differences from pairwise comparison (***p < 0.001; **p < 0.01, *p < 0.05).

Analysis of resistance revealed statistically significant strain and survival effects, as well as strain-by-survival interactions (*p* < 0.05; figure 2*a*). There was no statistically significant difference in resistance among the non-survivors, but all non-survivors (non-surviving BALB or A/J mice and C3H) ranked significantly lower in terms of resistance than any survivor (figure 2*a*). The mouse strains also differed significantly in tolerance to *Lm* infection, and tolerance varied between survival groups within the mouse strains (figure 2*b*). However, the ranking of the strains differed for the two traits (figure 2). In particular, non-survivors did not rank consistently lower in tolerance than survivors. C3H and B6J strains characterized by 0% and 100% survival, respectively, were at opposite ends of the resistance spectrum, but had similar tolerance estimates.

#### (ii) Dynamic trends in resistance and tolerance estimates

Although the actual resistance and tolerance estimates were sensitive to the timing of measurements, genetic variation in both resistance and tolerance could be detected throughout the entire 14-day infection period (electronic supplementary material, figure S2 and text S2). Ranking in both traits was relatively time stable, except for a reverse in ranking of A/J mice, which started the experiment as the most tolerant strain, and emerged as the least tolerant out of the three remaining mouse strains. B6J emerged as the most resistant mouse strain as early as 2 dpi, and eventually was also the most tolerant strain. In accordance with the results above, non-survivors differed significantly from survivors in resistance only, indicating that resistance may be more important than tolerance for survival of *Lm* infection.

### (b) Trajectory analysis

#### (i) Trajectory characteristics and determinants of survival

**Figure 3.**
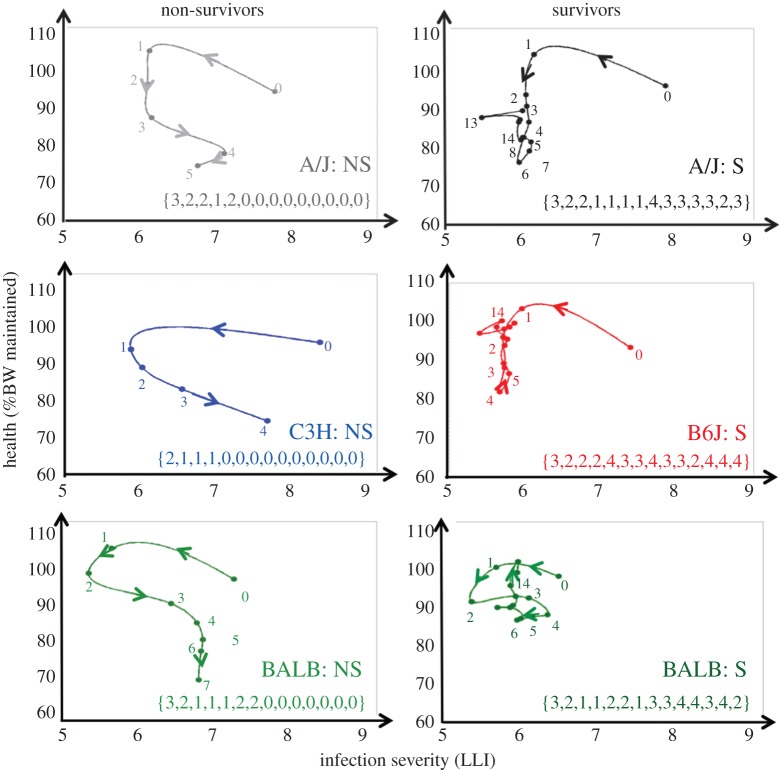
Representative trajectories for each mouse strain by survival outcome, together with the corresponding numerical sequence representing daily changes in infection severity and health (SH). We refer to figure 1 and the main text for explanation of the latter. The numbers in the trajectories denote the day at which the measurements were taken. ‘S’ and ‘NS’ denote survivors and non-survivors, respectively.

Visual inspection of individual trajectories (figure 3; electronic supplementary material, text S3) revealed common patterns in individuals' routes of infection and distinct survival characteristics. Four distinct phases over the course of infection were identified with characteristic changes in infection severity and health, as represented by different infection severity and health (SH) combinations (figure 3). Phase 1 described the establishment of infection during 0–1 dpi, and is related to the initial reduction in infection severity due to partial clearance of the inoculated pathogen accompanied by partial recovery in BW (i.e. S^−^H^+^). This phase was seen in all mice except C3H mice, most of which experienced weight loss (i.e. S^−^H^−^). Phase 2 corresponded to a period during which infection severity was stable but BW continued to drop (S^−^H^−^). Phase 3 was associated with resurgence in pathogen load and continued weight loss (i.e. S^+^H^−^). The final phase 4 differed between survivors and non-survivors. All survivors regained weight and controlled infection severity. Non-survivors, by contrast, continued to lose BW, although some were able to limit pathogen load. Mice in this phase generally fluctuated between expression of S^−^H^+^and S^+^H^+^. With the exception of phase 1, which lasted one day for all mice, the duration of the individual phases varied between mice. Only mice that survived the infection experienced an improvement in health (H^+^) at some stage after 4 dpi.

**Figure 4.**
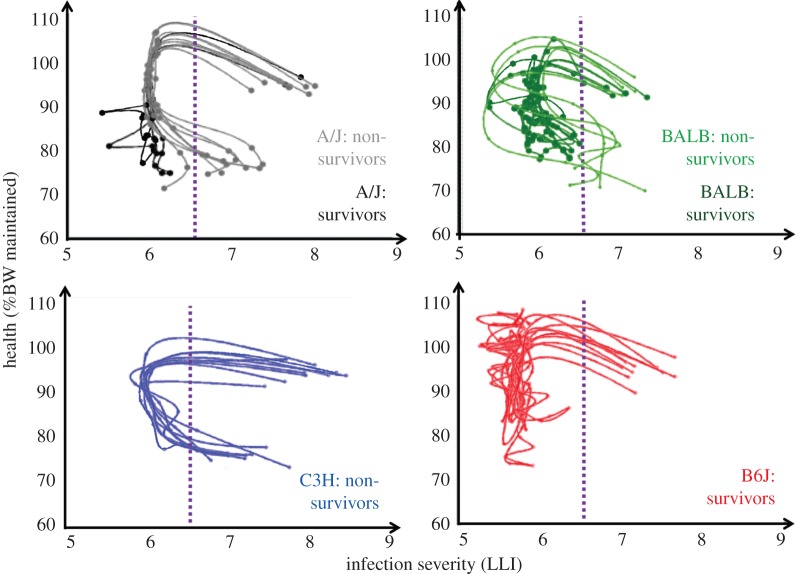
Spaghetti plots of individual trajectories of each mouse strain. The purple line shows the infection severity threshold that discriminates between survivors and non-survivors, independent of mouse inbred strain. All non-survivors except for one A/J mouse had crossed this threshold after 1 dpi, whereas none of the survivors had crossed this threshold after 1 dpi.

By overlaying trajectories, an infection severity threshold of approximately 6.5 LLI units for pathogen resurgence could be identified that discriminated between survival and death (figure 4). All mice that had crossed this threshold after 1 dpi succumbed to infection, regardless of their genotype, the exact day when the threshold was crossed (which occurred between 4 and 5 dpi) or whether infection severity temporarily decreased thereafter. All mice that suppressed pathogen replication below this threshold survived. Interestingly, there was no such discriminating threshold for BW loss within the limits of animal welfare regulations (figure 4). The results suggest that trajectories can provide more predictive thresholds for the definition of ethical experiment terminating endpoints than arbitrary cut-off values for BW losses.

#### (ii) Statistical analysis and genetic footprint of infection severity–health trajectories

Cluster analysis, combined with a permutation test, applied to the corresponding (truncated) trajectory sequences indicated that individual trajectories group into distinct trajectory types (electronic supplementary material, text S4). When the stipulated maximum number of clusters was two, the resulting clusters comprised either non-survivors or survivors. The permutation test confirmed a highly significant difference in the truncated SH sequences associated with both survival groups (*p* < 0.0001). Hence, infection paths of mice that succumbed to infection were significantly different to those of surviving mice at the early stage of infection.

**Figure 5.**
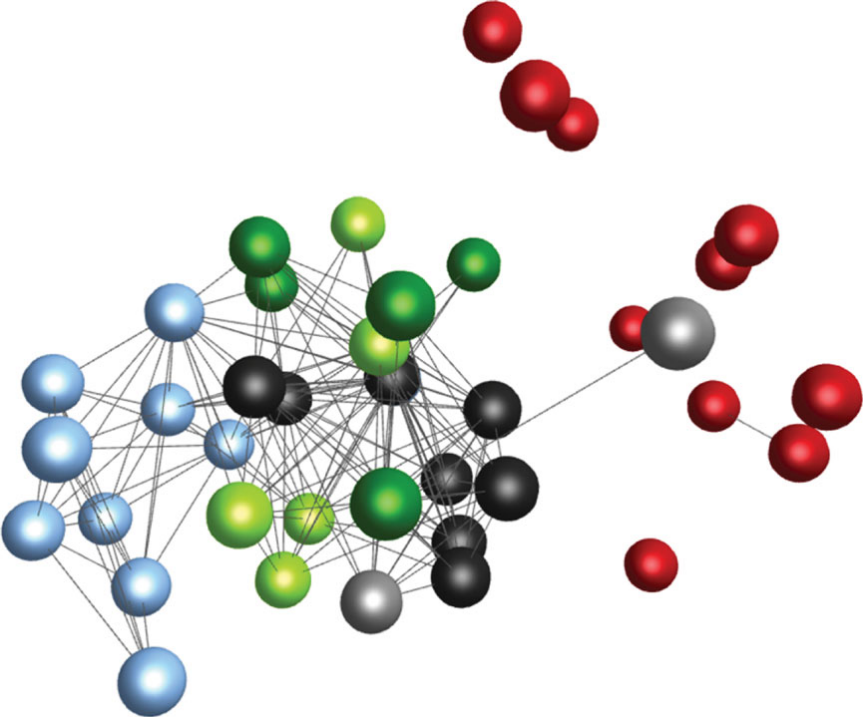
Graphical representation of similarities between truncated trajectory sequences associated with different mouse inbred strains and survival groups within the mouse strains. Each node (ball) represents an individual mouse, and each edge (connecting line) represents the degree of similarity between trajectory sequences of two mice, represented by 1−H, where H is the pairwise Hamming distance. Only similarities 1−H > 0.8 are depicted in the graph. Colours represent different mouse strains and survival groups, respectively. The graph was produced with BioLayout Express 3D software [24], which spatially distributes the nodes according to the similarity measure 1−H, so that individuals with similar trajectory sequences (i.e. 1−H close to 1) are placed in close proximity to each other, whereas individuals with different trajectory sequences (i.e. 1−H close to 0) are placed far apart. The graph illustrates that differences between trajectory sequences were on average smallest within each mouse strain, and greatest between C3H mice (light blue; all succumbing to infection) and B6J mice (red; all surviving infection). Within the A/J (black) and BALB (green) mice, sequences associated with survivors and non-survivors survival (survivors: light; non-survivors: dark) did not fall into different visual clusters. The graphical results were confirmed by statistical cluster analysis (see text).

When the stipulated maximum number of clusters was gradually increased, four distinct trajectory clusters (*p* < 0.02, for all cluster pairs) emerged, with the greatest sequence differences found between clusters comprising exclusively C3H and B6J mice, respectively (figure 5). The different clusters correspond to different survival outcomes and SH patterns within survivors/non-survivors, respectively, rather than to the four different inbred mouse strains (figure 5). However, each trajectory cluster is dominated by a specific mouse strain, suggesting that trajectories are partly genetically determined (figure 5). The permutation test applied to different mouse strains confirmed a statistically significant difference (*p* < 0.05) between trajectory sequences associated with different mouse strains, except for A/J and BALB mice (table 1). Interestingly, the trajectory sequences of surviving and non-surviving BALB or A/J mice were statistically indistinguishable (*p* = 0.53 and *p* = 0.06, respectively), implying that within a mouse strain trajectory sequences alone are insufficient for predicting survival outcome.

**Table 1.** *p*-values of permutation test used to assess whether mouse strains differ significantly in their SH trajectory sequences.

between-strain	A/J	C3H	B6J	BALB
A/J		<10^−6^	0.0003	0.31
C3H			<10^−6^	0.0002
B6J				0.0002
BALB				.

## 4. Discussion

Host genetic variation in both resistance and tolerance can account for a substantial part of the observed variation in host response to infection [2,7,10,25]. Many studies have provided conclusive evidence for genetic variation in host resistance to *Lm* [2,26–29]*.* We have demonstrated that mice from genetically distinct inbred strains, previously found to differ significantly in resistance to the bacteria, also differ in tolerance. By convention, resistance and tolerance are considered as static traits that constitute alternative host defence strategies against invading pathogens [2,3]. The data show clearly that expression of resistance (reduction in pathogen load) and tolerance (damage prevention and repair) and their relative contribution to survival vary over the time course of infection. We therefore propose a paradigm shift in considering resistance and tolerance as dynamic, rather than static traits. In practice, this can only be achieved through time series measurements in individual infected hosts, which in turn depend upon non-invasive technologies, such as imaging. Recently, there have been rapid advances in the development of such imaging technologies [30–32], and this has led to increasing demands for advanced statistical tools to analyse infection dynamics, such as the trajectory methods proposed here.

The novelty of this research lies in the development of simple and versatile mathematical tools for capturing the dynamic development of resistance and tolerance in each individual, and their relative importance on the outcome of infection. The conventional reaction-norm approach to tolerance has severe limitations that have hampered progress in tolerance studies [11]. First, it usually restricts tolerance estimates to group level, which is not helpful for improving tolerance of individuals or identifying tolerance genes. Second, the high data demand associated with estimating tolerance parameters from this approach limits reaction norms to linear models, thus ignoring all biological understanding of the highly nonlinear and time-dependent relationship between pathogen burden and health [13]. By contrast, individual trajectories, which can be easily constructed if longitudinal measurements are available, illustrate how changes in infection severity are related to health change within each individual throughout infection. Although not synonymous with resistance and tolerance, the two-dimensional trajectory vectors crudely reflect how resistance and tolerance are coexpressed at different stages of infection. For example, simultaneous decrease in infection severity and health (S^−^H^−^) reflects expression of resistance at the cost of deterioration in health, indicating incomplete tolerance. As demonstrated in this study, trajectories can reveal distinct phases of infection associated with different patterns of coexpression of resistance and tolerance, and illustrate for each individual the two-dimensional path towards death or survival. Previous studies of trajectories have defined ‘bad neighbourhoods’ in the infection-severity health plane that appear predictive for fatal infection outcome [2,12,15]. In the mouse data, we identified an infection severity threshold that discriminated between death and survival (figure 4). All mice that succumbed to the infection experienced a drastic increase in infection severity between 3 and 7 dpi, whereas all survivors managed to restrict pathogen resurgence below this threshold during this critical phase. The critical infection severity threshold was independent of host genotypes and timing. Any mouse that crossed this threshold eventually succumbed to infection, even if thereafter it managed to reduce infection severity below the threshold. Interestingly, there was no apparent health threshold that discriminated between death and survival. All mice experienced BW loss as a consequence of infection, but all the survivors and none of the non-survivors managed to recover some of the lost weight, in some cases despite continued increase in pathogen load. Our results thus indicate that both early expression of resistance and tolerance at the later stages of infection are important determinants of survival to *Lm* infections. Discriminatory thresholds, such as those identified here, can provide more informative criteria than BW for defining humane endpoints for termination of animal infection.

Individual trajectories have been used previously to classify and predict host responses to infection, but their assessment was limited to qualitative analysis [14,15]. By transforming visual trajectories into numerical sequences that preserve the key topological trajectory features, we were able to subject trajectories to rigorous statistical analysis. Our statistical analysis confirmed that individual trajectories cluster into a limited number of genetically regulated distinct trajectory types [14,15]. Trajectories thus open new avenues for genetic studies of host response to infections. Future studies may focus on genetic dissection of different trajectory types to identify novel genetic variants that control infection dynamics at a molecular level.

Despite a clear genetic footprint in trajectory patterns, trajectory sequences could not capture within-strain differences in survival outcome. This could be due to several reasons: first, BW may only be a crude indicator of health [2]. Alternatively, as mice were euthanized due to welfare considerations based upon weight loss, some of the mice classified as non-survivors may actually have survived the infection. Furthermore, survival outcome may be partly determined by individual differences in the gastro-intestinal flora, which have been found to show substantial inter-strain variation even in similarly highly controlled environments as used in our study [33]. Higher-dimensional trajectories comprising other types of measurements (e.g. related to the immune response or microbiota) in addition to measures of health and pathogen load may shed light on relevant host response mechanisms controlling an individual's infection path and its outcome. Note that, although more difficult to visualize, multi-dimensional trajectories can still be represented as a series of vectors defined by their direction and length, and are thus amenable to similar statistical analyses as those presented here.

Previous studies have estimated an antagonistic relationship between resistance and tolerance at the phenotypic and genetic level [7,34]. These results led to the notion of a trade-off between resistance and tolerance mechanisms, and their consideration as alternative host defence strategies to fight infections, and shaped predictions of evolutionary consequences for both hosts and pathogens [35–38]. These studies do not take account of the dynamic relationships that emerge from our study. For example, expression of resistance at the early stages of infection is likely to affect the expression of tolerance at the later stages as fast pathogen clearance may prevent tissue damage and obviate any requirement for damage prevention or repair mechanisms associated with tolerance. Conventional statistical models that do not account for this kind of interdependence between traits may produce a spurious antagonistic relationship between traits, on both the phenotypic and genetic levels [39], even if resistance and tolerance are controlled by different sets of genes or genetic pathways as suggested by immunological evidence [1,4]. Trajectories enable us to bypass the complex relationship between resistance and tolerance, and may give rise to novel phenotypes for future genetic analyses that may lead to the discovery of genes that control an individual's infection path.

All of the infected mice experienced a substantial BW loss after the initial reduction in pathogen burden, but of itself, BW loss did not influence survival. From a resource allocation perspective, this would suggest that resistance mechanisms are costly [40,41]. By reducing resources allocated to other functions, such as searching for and digesting food, the host may be able to direct resources to the immune response, resulting in temporary BW loss. This has been put forward as the evolutionary basis of pathogen-induced anorexia and ‘sickness’ behaviours [40,42–44]. Our previous applications of the resource allocation theory to assess the effect of genetic resistance on the long-term effects of infection, showed that hosts with greater genetic resistance may suffer greater performance loss (e.g. growth or BW loss) in the short term, but are able to revert to original levels of performance faster than non-resistant genotypes [45]. This is consistent with our study, where the B6J mice emerged as most resistant genotype after 3 dpi and were the only mouse strain that managed to fully restore the original BW within 14 dpi.

To study the dynamic coexpression patterns of resistance and tolerance to *Lm* in different mouse inbred strains, we have taken advantage of a bioluminescent *Lm* infection model in which the listerial strain EGDe-InlA-mur-lux recognizes the host receptor E-cadherin and intestinal expressed N-cadherin [22,23,46,47]. Other *Lm* infection models have been shown to elicit different host responses [47,48]. It would be interesting to apply our novel methods to data from these models to determine whether the dynamic contributions of resistance and tolerance to survival are preserved across different pathogen and host strains. However, this would require the introgression of humanized alleles of *CDH1* (encoding E-cadherin) into the different mouse genetic backgrounds followed by repeated backcrossing to make mice permissive to oral *Lm* challenge [22,26].

In conclusion, our study complements existing evidence for genetic variation in both host resistance and tolerance, and for the importance of both host strategies in fighting infections [1–9,21,45]. However, our study also highlights the potential benefits that may arise from considering the dynamic patterns of coexpression of genetic resistance and tolerance over the time course of infection. Trajectories capture the dynamic signature and genetic footprint of both mechanisms on the level of individuals, together with their impact on survival.

## Data accessibility

The datasets supporting this article have been uploaded as part of the electronic supplementary material, text S6.
